# Catalpol Protects Against Pulmonary Fibrosis Through Inhibiting TGF-β1/Smad3 and Wnt/β-Catenin Signaling Pathways

**DOI:** 10.3389/fphar.2020.594139

**Published:** 2021-01-29

**Authors:** Fan Yang, Zhen-feng Hou, Hao-yue Zhu, Xiao-xuan Chen, Wan-yang Li, Ren-shuang Cao, Yu-xuan Li, Ru Chen, Wei Zhang

**Affiliations:** ^1^ College of Traditional Chinese Medicine, Shandong University of Traditional Chinese Medicine, Jinan, China; ^2^ College of Life Sciences, Shandong Normal University, Jinan, China; ^3^ School of Public Health, Xiangya Medical College, Central South University, Changsha, China; ^4^ Second School of Clinical Medicine, Beijing University of Chinese Medicine, Beijing, China; ^5^ Biomedical Research Institute of Fudan University, Shanghai, China; ^6^ Department of Pulmonary Diseases, Affiliated Hospital of Shandong University of Traditional Chinese Medicine, Jinan, China

**Keywords:** catalpol, idiopathic pulmonary fibrosis, epithelial-mesenchymal transition, Smad3, Wnt3a

## Abstract

Idiopathic pulmonary fibrosis (IPF) is a fatal lung disease characterized by fibroblast proliferation and extracellular matrix remodeling; however, the molecular mechanisms underlying its occurrence and development are not yet fully understood. Despite it having a variety of beneficial pharmacological activities, the effects of catalpol (CAT), which is extracted from *Rehmannia glutinosa*, in IPF are not known. In this study, the differentially expressed genes, proteins, and pathways of IPF in the Gene Expression Omnibus database were analyzed, and CAT was molecularly docked with the corresponding key proteins to screen its pharmacological targets, which were then verified using an animal model. The results show that collagen metabolism imbalance, inflammatory response, and epithelial-mesenchymal transition (EMT) are the core processes in IPF, and the TGF-β1/Smad3 and Wnt/β-catenin pathways are the key signaling pathways for the development of pulmonary fibrosis. Our results also suggest that CAT binds to TGF-βR1, Smad3, Wnt3a, and GSK-3β through hydrogen bonds, van der Waals bonds, and other interactions to downregulate the expression and phosphorylation of Smad3, Wnt3a, GSK-3β, and β-catenin, inhibit the expression of cytokines, and reduce the degree of oxidative stress in lung tissue. Furthermore, CAT can inhibit the EMT process and collagen remodeling by downregulating fibrotic biomarkers and promoting the expression of epithelial cadherin. This study elucidates several key processes and signaling pathways involved in the development of IPF, and suggests the potential value of CAT in the treatment of IPF.

## Introduction

Pulmonary fibrosis (PF), which usually manifests at the end stages of various interstitial lung diseases, is characterized by alveolar epithelial cell damage and abnormal deposition of extracellular matrix (ECM) (Herrera et al., 2018). There are a variety of causes of PF—idiopathic pulmonary fibrosis (IPF) is a form of an unexplained and severe PF with a 5-years survival rate of less than 30% (Nalysnyk et al., 2012). Currently, the only drugs recommended for the treatment of mild-to-moderate IPF are pirfenidone (PFD) and nintedanib, both of which, however, fail to prolong the survival of patients (Ogura et al., 2015; Jo et al., 2016). Moreover, PFD has several side effects, such as gastrointestinal reactions, rash, and photosensitivity (Cottin and Maher, 2015). Thus, there is an urgent need for the development of new drugs for IPF.

Bioinformatics utilizes sequence comparison and cluster analysis methods to extract biological information using technologies such as GeneChip, which enables more comprehensive and systematic study of disease pathology. With the development of high-throughput microarray and sequencing technologies in recent years, it has become possible to investigate the gene expression profiles of IPF and the corresponding changes in PF tissue and key genes. Moreover, the differentially expressed gene (DEG) data obtained in this manner has in many cases enabled the successful screening of potential drugs by the docking of small molecular compounds with the corresponding proteins.

*Rehmannia glutinosa* is a traditional Chinese herb that has been widely used for the treatment of circulatory diseases for thousands of years (Li and Kan, 2017; Xu et al., 2017). Catalpol (CAT), a compound extracted from *R. glutinosa*, is known to have anti-inflammatory, anti-epithelial-mesenchymal transition (EMT), anti-oxidative, anti-apoptoti, and anti-angiogenesis properties, and to have favorable pharmacological effects in patients with asthma (Chen et al., 2017), lung cancer, glomerulonephritis, and colon cancer (Zhu et al.,2017b; Yang et al., 2020). In recent years, studies have focused on the protective effects of R. glutinosa extract, represented by CAT, on nerve cells and kidney cells. The mechanism of action is to target SIRT1 and Wnt/β-catenin signaling pathways, stabilize cytoskeleton and enhance autophagy (Chen et al., 2019; Liu et al., 2019; Zhang et al., 2019; Zhou et al., 2019; Cheng et al., 2020; Wang et al., 2020). However, its effects on IPF remain unknown. In order to investigate the effect of CAT on IPF, we screened the Gene Expression Omnibus (GEO) database for DEGs in IPF and investigated the important signaling pathways and proteins involved in the development of IPF. We also used molecular docking to virtually screen proteins expressed by the DEGs and estimate the theoretical stability of their binding to CAT. Furthermore, we established a rat IPF model to verify the efficacy of CAT against IPF and explore its mechanisms of action.

## Materials and Methods

### Microarray Data

The following gene expression profile datasets were obtained from the GEO database (https://www.ncbi.nlm.nih) (Rosas et al., 2008; Meltzer et al., 2011; DePianto et al., 2015): GSE10667 (platform: GPL4133 Agilent-014850 Whole Human Genome Microarray 4 × 44K G4112F), obtained using a total of 31 IPF and 15 normal specimens; GSE24206 (platform: GPL570 Affymetrix Human Genome U133 Plus 2.0 Array), obtained using 17 IPF and six normal specimens; and GSE53845 (platform: GPL6480 Agilent-014850 Whole Human Genome Microarray 4 × 44K G4112F), obtained using 40 IPF and eight normal specimens.

### Data Processing

GEO2R allows users to compare different sample groups in the GEO series and to screen genes that are differentially expressed under different experimental conditions. GEO2R compares original submitter-supplied processed data tables using the GEOquery and limma (Linear Models for Microarray Analysis) R packages from the Bioconductor project. The GEOquery R package parses GEO data into R data structures that can be used by other R packages. The limma R package has emerged as one of the most widely used statistical tools for identifying DEGs. We used the GEO2R online software to analyze the microarray data provided by the original submitter and identify DEGs with recognition thresholds set to false discovery rate (FDR) < 0.05 and |log2 fold change (FC)| > 1. The upregulated and downregulated genes were analyzed, and volcano maps of the three datasets were drawn. We then selected the DEGs present in two or three datasets as the total differential genes, which exceeded 600 in number. Venn diagrams were drawn for upregulated and downregulated genes. Finally, hierarchical cluster analysis was performed on DEGs, with heat maps drawn for the three chips using the heatmap package.

### Gene Ontology and Pathway Enrichment Analysis

DAVID (http://david.abcc.ncifcrf.gov/) is an online gene function annotation tool that provides information regarding the biological significance of a large number of genes (Huang et al., 2007). The GO analysis provided by DAVID for researchers includes the cellular components (CC), molecular functions (MF), and biological processes (BP) categories (Gene Ontology, 2006). We used this database for annotation, data analysis, and visualization. In addition, we performed a Kyoto Encyclopedia of Genes and Genomes (KEGG) pathway function enrichment analysis on the DEGs described above (Kanehisa and Goto, 2000). *p* < 0.05 was considered statistically significant.

### Protein-Protein-Interaction Network Construction and Module Analysis

PPI analysis can be used to directly explain the molecular mechanism underlying key protein interactions. In this study, we used the STRING database to construct PPI networks for all upregulated and downregulated proteins (Missiuro et al., 2009). Then, we utilized Cytoscape for visualization and the MCODE plug-in for subset analysis (Barabasi et al., 2011).

### Molecular Docking

Based on the differential expression results of the above genes and proteins and information from the pathway enrichment analysis, core proteins were selected for forward molecular docking with CAT. CAT molecular structure data were obtained from the PubChem website (https://pubchem.ncbi.nlm.nih.gov/) (Kim et al., 2019) and protein crystal structure data from the RCSB website (http://www.rcsb.org/) (Berman et al., 2003). We used Discovery Studio 2016 (DS) for creating the 2D and 3D effect pictures and for the molecular docking calculations. DS was used to extract the ligands with crystal structure of protein. After exposing active sites, excluding crystalized water, hydrogens, and side chain residues, the CHARMm force field and Momany-Rone charge were added. The docking file of the active region was then obtained using default parameters. Docking files for crystal structures without ligand were automatically generated using default parameters. Using the DS CDOCKER module, the possible multiple conformations, interaction energies and main action sites of CAT to target protein docking were obtained. Molecular docking results can indicate the mechanism of action of CAT on IPF and guide the selection of related detection indicators in subsequent animal experiments.

### Antibodies and Reagents

CAT (purity: 98.3%, HPLC) was purchased from Shanghai Yuanye Bio-Technology Co., Ltd. (L20N8Y48597), bleomycin (BLM) from Cool Chemical Technology Co., Ltd. Beijing (S656455V), and PFD from Beijing Continent Pharmaceutical Co., Ltd. (190806). The Masson tricolor staining kit (G1006-100), hematoxylin-eosin (HE) staining kit (GP1031), reactive oxygen species (ROS) test kit (2019-07), detection kits for hydroxyproline (HYP) (201900711), aspartate aminotransferase (AST) (20190712), alanine aminotransferase (ALT) (20190711), malondialdehyde (MDA) (20190830), superoxide dismutase (SOD) (20191125), and the bicinchoninic acid (BCA) method total protein quantification kit (20190711) were all purchased from Nanjing Jiancheng Bioengineering Research Institute. Rabbit anti-Wnt3a antibody (BS-1700r), rabbit anti-phosphorylated-Smad3 antibody (bs-19452r), and rabbit anti-Smad3 antibody (BS-3484r) were purchased from Biosynthesis Biotechnology Inc. Beijing, China. Rabbit anti-α-SMA antibody (GB11044), rabbit anti-matrix metalloprotease (MMP)-7 antibody (A0695), rabbit anti-COL1A1 antibody (GB11022-3), rabbit anti-COL3A1 antibody (GB13023-2), rabbit anti-β-catenin antibody (GB12015), rabbit anti-GSK-3β antibody (GB11099), rabbit anti-E-cadherin (E-cad) antibody (GB13083), and HRP-labeled goat anti-rabbit IgG antibodies (GB23303) were purchased from Wuhan Servicebio Technology Co., Ltd. The rat TGF-β1 enzyme-linked immunosorbent assay (ELISA) kit (E04019240) was purchased from Cusabio Biotech Co., Ltd. Wuhan. Rabbit anti-anti-β-actin antibody (AC026), rabbit anti-phosphorylated-GSK3β antibody (AP0039), and rabbit anti-phosphorylated-β-catenin antibody (AP0979) were obtained from ABclonal Biotechnology Co., Ltd. Wuhan. Rat IL-6 (CSB-E04640r), IL-1β (CSB-E08055r), and TNF-α (CSB-E11987r) ELISA kits were purchased from Wuhan Huamei Bioengineering Co., Ltd.

### Animal Grouping and Modeling

The study protocol was approved by the Research Ethics Committee of the Affiliated Hospital of Shandong University of Traditional Chinese Medicine (Approval No. AWE-2019-046) and followed the National Institutes of Health Guide for the Care and Use of Laboratory Animals (NIH Publications No. 8023, revised 1978). Male Sprague Dawley rats (180–220 g, SPF grade) purchased from Jinan Pengyue Experimental Animal Breeding Co., Ltd. (Certificate No. SCXK [Lu]2014-0007, Jinan, China) were maintained under 12-h light/12-h darkness conditions with free access to feed and water. After 7 days of adaptive breeding, the rats were randomly divided into six groups (6 in each group): 1) saline (NS) group; 2) BLM + NS group; 3) BLM + CAT (10 mg/kg/d) group; 4) BLM + CAT (20 mg/kg/d) group; 5) BLM + CAT (40 mg/kg/d) group; and 6) BLM + PFD (150 mg/kg/d) group. A single intratracheal instillation of BLM (5 mg/kg) was used to induce PF in rats. After the modeling, rats in the CAT groups were injected intraperitoneally with the corresponding concentration of drugs, while rats in the PFD group were administered PFD intragastrically; all animals were sacrificed 28 days later. Rat blood from the abdominal aorta was centrifuged at 5,000 rpm for 10 min at 4°C, and the serum was stored at -80°C. Lung tissues were also collected and weighed. Lung index was calculated as lung weight (g)/(body weight (g) × 100%. The whole lung was lavaged three times using 2 ml of physiological saline, and bronchoalveolar lavage fluid (BALF) was then collected. Some of the lung tissue was placed in 4% paraformaldehyde, with the rest frozen in liquid nitrogen and stored at −80°C for further examination.

### Morphological and Histological Analysis

Lung tissues fixed using 4% paraformaldehyde for 48 h were embedded in paraffin and sectioned (thickness, 5 μm). The slices were stained with HE and Masson trichrome for the evaluation of lung tissue pathological changes and then imaged at a magnification of ×200 using an optical microscope. According to the Szapiel scoring standard and Ashcroft scoring standard, the degree of alveolitis and PF were scored, respectively (Szapiel et al., 1979; Ashcroft et al., 1988).

### Measurement of HYP, MDA, and ROS Levels and SOD, ALT, and AST Activity

Lung tissues were ground in cold physiological saline to obtain a 10% homogenate, which was centrifuged at 3,500 rpm for 10 min at 4°C, and the supernatant was retained for the assessing HYP, MDA, and ROS levels and SOD activity. Serum samples were used to detect ALT and AST activity according to the corresponding kit instructions.

### ELISA

BALF and serum were prepared for ELISA, and the appropriate kits were used to assess the TNF-α, IL-1β, and IL-6 levels in rat BALF and TGF-β1 level in serum samples.

### Western Blotting

Lung tissues were harvested, washed with cold PBS and lysed with PIRA lysis buffer containing protease and phosphatase inhibitor and phenylmethylsulphonyl fluoride for 30 min on ice and then were centrifuged at 12,000 g for 15 min at 4°C; thereafter, proteins were extracted from the lung tissue lysates and the BCA protein detection kit was used to measure the protein concentrations according to the instructions provided. Equal amounts of protein were separated by sodium dodecyl sulfate-polyacrylamide gel electrophoresis and transferred onto polyvinylidene fluoride membranes. After blocking with 5% (wt/vol) nonfat milk in Tris-buffered saline Tween-20 at room temperature, the membranes were incubated with primary antibodies against α-SMA (diluted 1:2000), COL1A1 (diluted 1:1,000), COL3A1 (diluted 1:750), *p*-Smad3 (diluted 1:750), Smad3 (diluted 1:1,000), Wnt3a (diluted 1:1,000), p-β-catenin (diluted 1:1,000), β-catenin (diluted 1:1,200), *p*-GSK-3β (diluted 1:1,000), or GSK-3β (diluted 1:1,000) overnight at 4°C. After washing four times with TBST, they were incubated with goat anti-rabbit secondary antibody for 1.5 h at room temperature and then washed again four times with TBST. The protein bands on the membrane were then visualized using an enhanced chemiluminescence reagent. Band intensities were quantified using ImageJ.

### Immunohistochemical Analysis

After dewaxing, the lung slices were subjected to antigen recovery in citrate buffer under microwave heating. The slices were cooled down to room temperature, blocked with 3% bovine serum albumin for 30 min, and then incubated with the following primary antibodies overnight at 4°C as appropriate: rabbit anti-α-SMA antibody (diluted 1:1,000), anti-COL1A1 antibody (diluted 1:1,000), anti-COL3A1 antibody (diluted 1:200), anti-Smad3 antibody (diluted 1:200), anti-Wnt3a antibody (diluted 1:250), anti-β-catenin antibody (diluted 1:200), anti-GSK-3βantibody (diluted 1:600), anti-E-cad antibody (diluted 1:400), and anti-MMP-7 antibody (diluted 1:100). The slices were then washed with phosphate-buffered saline (PBS) and incubated with goat anti-rabbit secondary antibody (diluted 1:200) at 37°C for 50 min. After rinsing with PBS, the slices were stained with diaminobenzidine and counterstained with hematoxylin. The average optical density was measured using ImageJ.

### Statistical Analysis

Data are represented as mean ± standard deviation (SD). Differences between the groups were evaluated using one-way analysis of variance followed by the least significant difference (LSD) post hoc test. A value of *p* < 0.05 was considered statistically significant. IBM SPSS Statistics 19.0 (IBM SPSS Software, NY, United States) and GraphPad Prism Version 8.0 (GraphPad Software, San Diego, CA, United States) were used for statistical analyses and figure preparation.

## Results

### Analysis of DEGs in IPF

**FIGURE 1:**
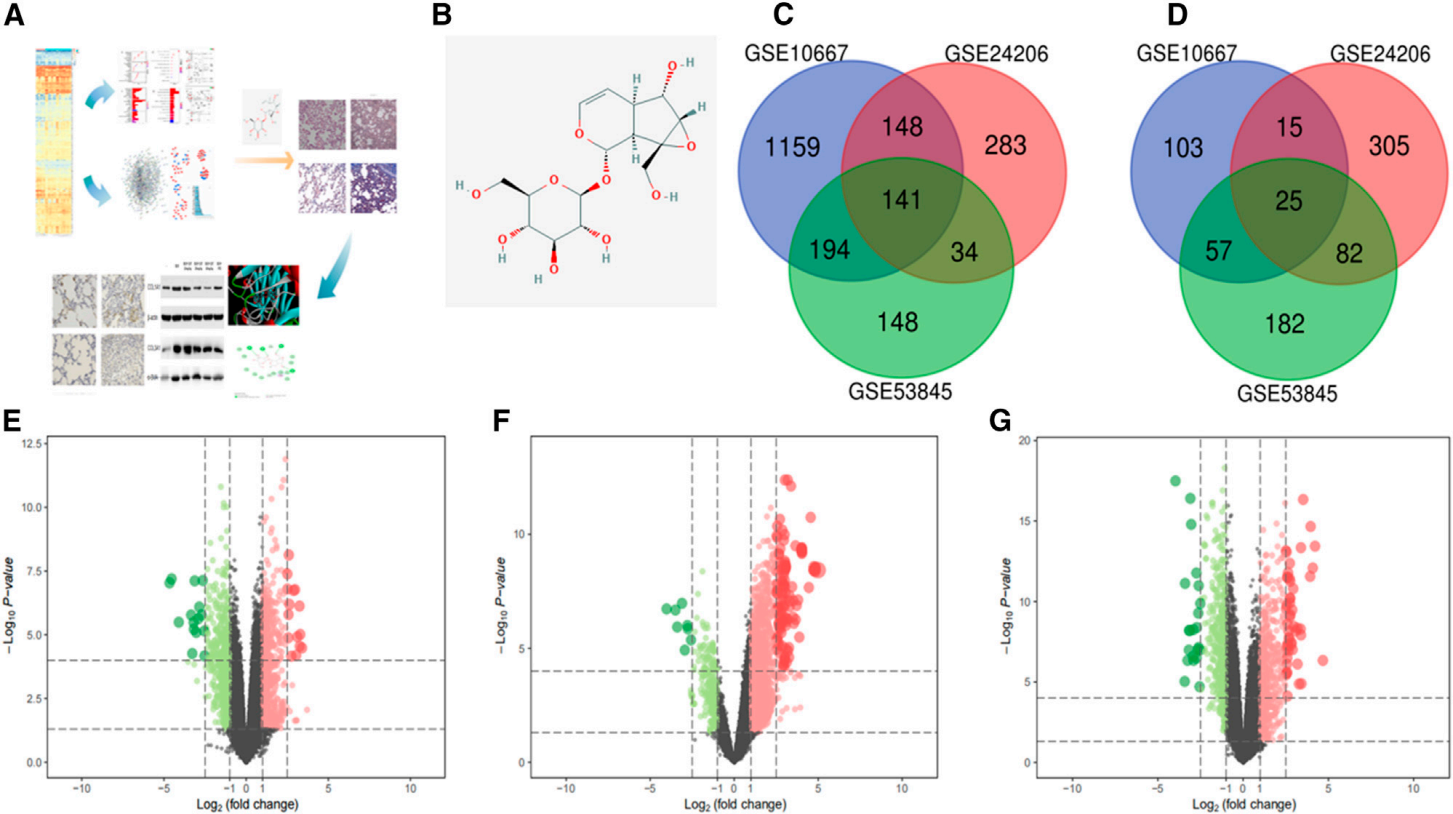
In this study, we screened the GEO database for DEGs in IPF to find the key signaling pathways and proteins in the occurrence and development of this disease. Next, molecular docking was used to virtually screen the DEG proteins and estimate the theoretical stability of their binding to CAT. Furthermore, a rat IPF model was established to verify the effect of CAT on the key signaling pathways in IPF and to explore its mechanism of action **(A)**. The chemical structure of CAT **(B)**. Upregulated genes in the datasets **(C)**. Downregulated genes in the datasets **(D)**. **(E–G)** are the volcano maps and heat maps of the highly expressed genes and the low expressed genes in the data sets GSE24206, GSE10667, and GSE53845. In the volcano map, green represents down-regulated genes and red represents up-regulated genes; in the heat map, blue represents down-regulated genes, and red represents up-regulated genes between IPF samples and normal samples. *p* < 0.01 and FC > 1 were considered as cut-off values.

The study flow chart is shown in Figure 1A. A total of 517 genes were upregulated in the three data sets during the IPF process, while 179 genes were downregulated. The numbers of upregulated and downregulated genes are shown in Figures 1C,D. The volcano map and heat map of 141 high expression genes and 25 low expression genes in GSE24206 are shown in Figure 1E. Among all up- or downregulated genes, the top five with the most significant changes in differential expression were ZMAT, CRIP, PSD, FNDC1 (upregulated), and BTNL8 (downregulated). Their functions are mainly related to cell cycle regulation and EMT. For example, ZMAT is a p53 target gene that regulates the cell cycle and apoptosis (Bersani et al., 2014). FNDC1 is ubiquitous in the cell matrix, and related membrane receptors and enzymes can mediate Cx43 phosphorylation and G protein signal transduction to regulate cell permeability and apoptosis (Sato et al., 2009). GSK-3β is an important signal transduction molecule in the Wnt/β-catenin signaling pathway, and CRIP1 promotes EMT through zinc-induced *p*-GSK-3β in colorectal cancer (He et al., 2019). The downregulation of BTNL8 expression is related to excessive inflammation and destruction of epithelial tissue integrity (Mayassi et al., 2019). The DEGs in GSE10667 and GSE53845 are shown in Figures 1F,G and Supplementary Figures S1-S6.

### GO Function Enrichment Analysis, KEGG Pathway Analysis, and PPI Network Analysis

**FIGURE 2:**
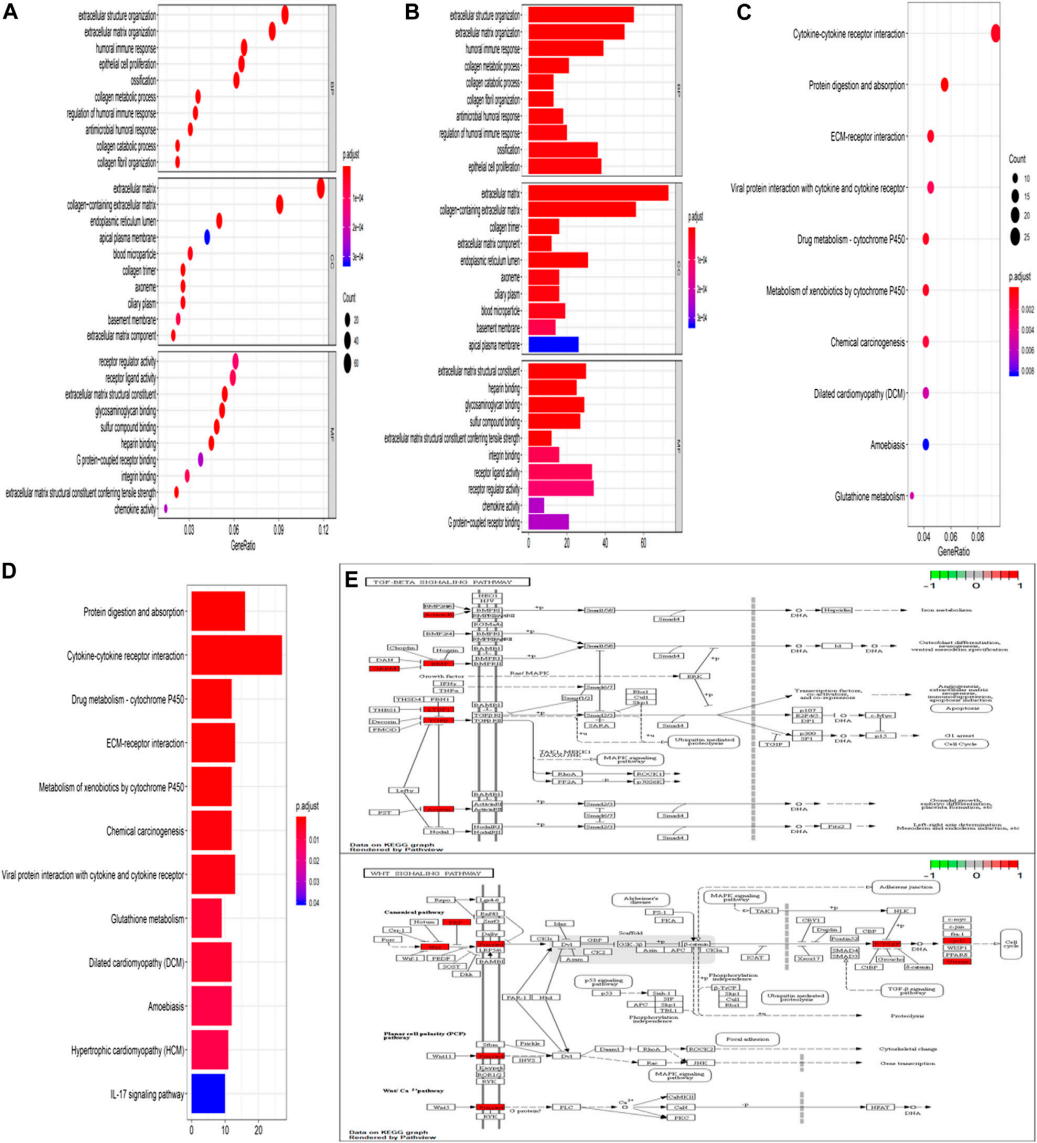
GO function enrichment analysis and KEGG pathway analysis. Bubble chart of enrichment analysis results of GO function **(A)**. Histogram of enrichment analysis results of GO function **(B)**. Bubble chart of KEGG pathway enrichment analysis results **(C)**. Histogram of KEGG pathway enrichment analysis results **(D)**. The key target of the TGF-β/Smad signaling pathway in IPF **(E)**. The key target of the Wnt signaling pathway in IPF **(F)**.

**FIGURE 3:**
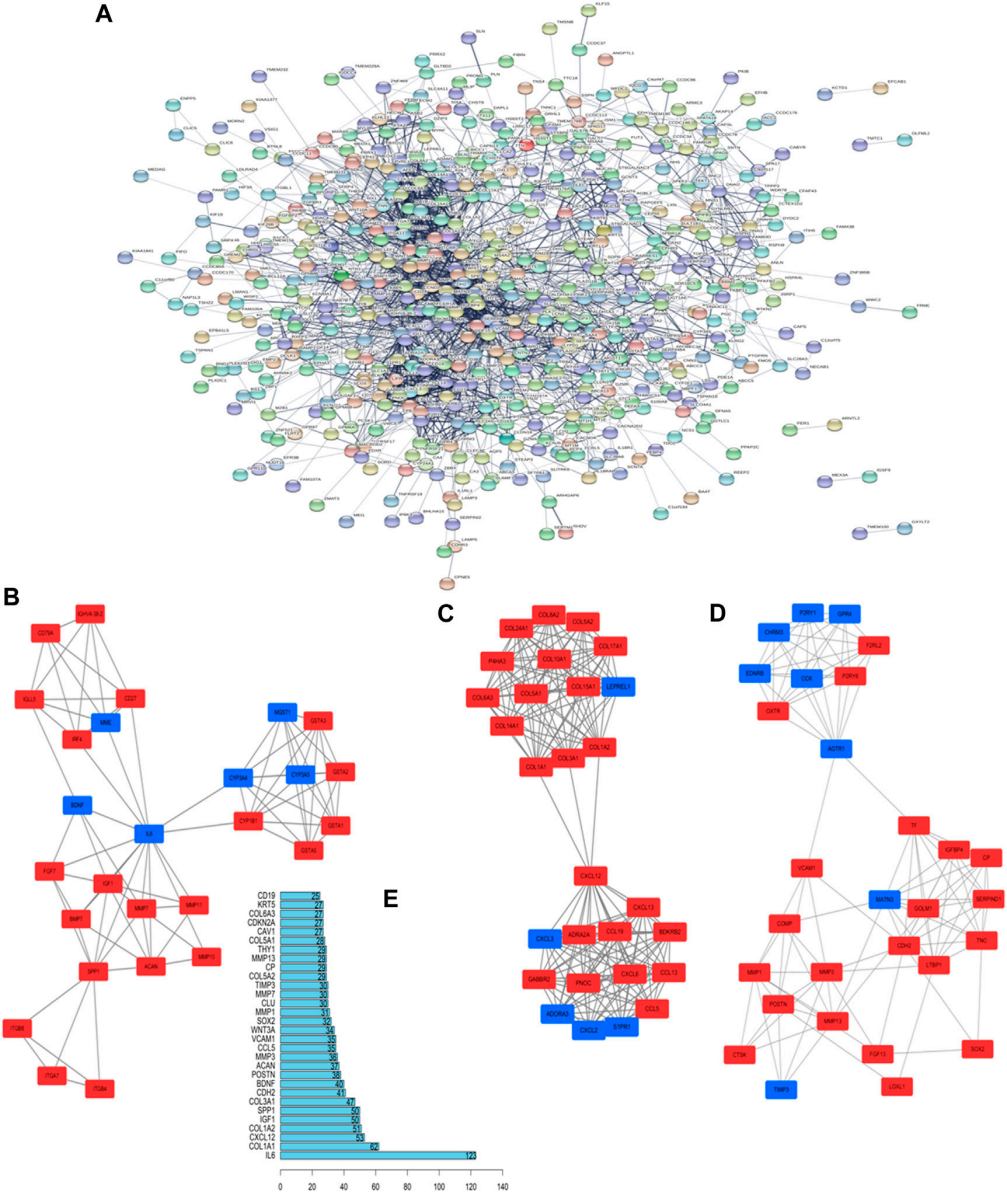
Key proteins and interactions in IPF. **(A)** PPI network of differentially expressed proteins in IPF. This network consists of 572 nodes and 2,432 edges. **(B–D)** The three important core modules in MCODE module analysis. The red square represents upregulation and the blue block represents downregulation. **(E)** Ranking of correlation degree of key proteins.

GO analysis confirmed that the DEGs in IPF mainly coded for proteins involved in the ECM environment and the collagen metabolism process (Figures 2A,B), which was also evidenced by the CC and MF enrichment analyses. The BP analysis suggested that the humoral immune response of IPF patients is unbalanced and is characterized by the high expression of inflammatory mediators. Correspondingly, KEGG analysis (Figures 2C,D) showed high confidence in the protein degradation and synthesis process and the interaction between cytokines and receptors. It has been reported that the Wnt signaling pathway is activated in IPF, and the regulation of EMT by it is an important biological process in IPF. The TGF-β signaling pathway is a key pathway for M2 macrophages to induce EMT (Zhu et al., 2017a; Ko et al., 2019) and can also promote the development of IPF by altering the 3’-UTR of target mRNAs. The key signal transduction molecules in the two signaling pathways are shown in Figure 2E,F. An IPF-related PPI network (Figure 3A) was constructed based on the STRING database. The ranking of the top 30 key proteins is shown in Figure 3E and Supplementary Table S1. The top five pivot proteins were IL-6, COL1A1, CXCL12, COL1A2, and IGF1 (PPI enrichment *p*-value:< 1.0e-16). MCODE module analysis (Figures 3B–D) showed that chemokine signal transduction, cell cycle and proteasome, and collagen and vascular remodeling were the main functions of the three important core modules, suggesting that the expression of the MMP family was upregulated.

### CAT Improves BLM-Induced PF

**FIGURE 4:**
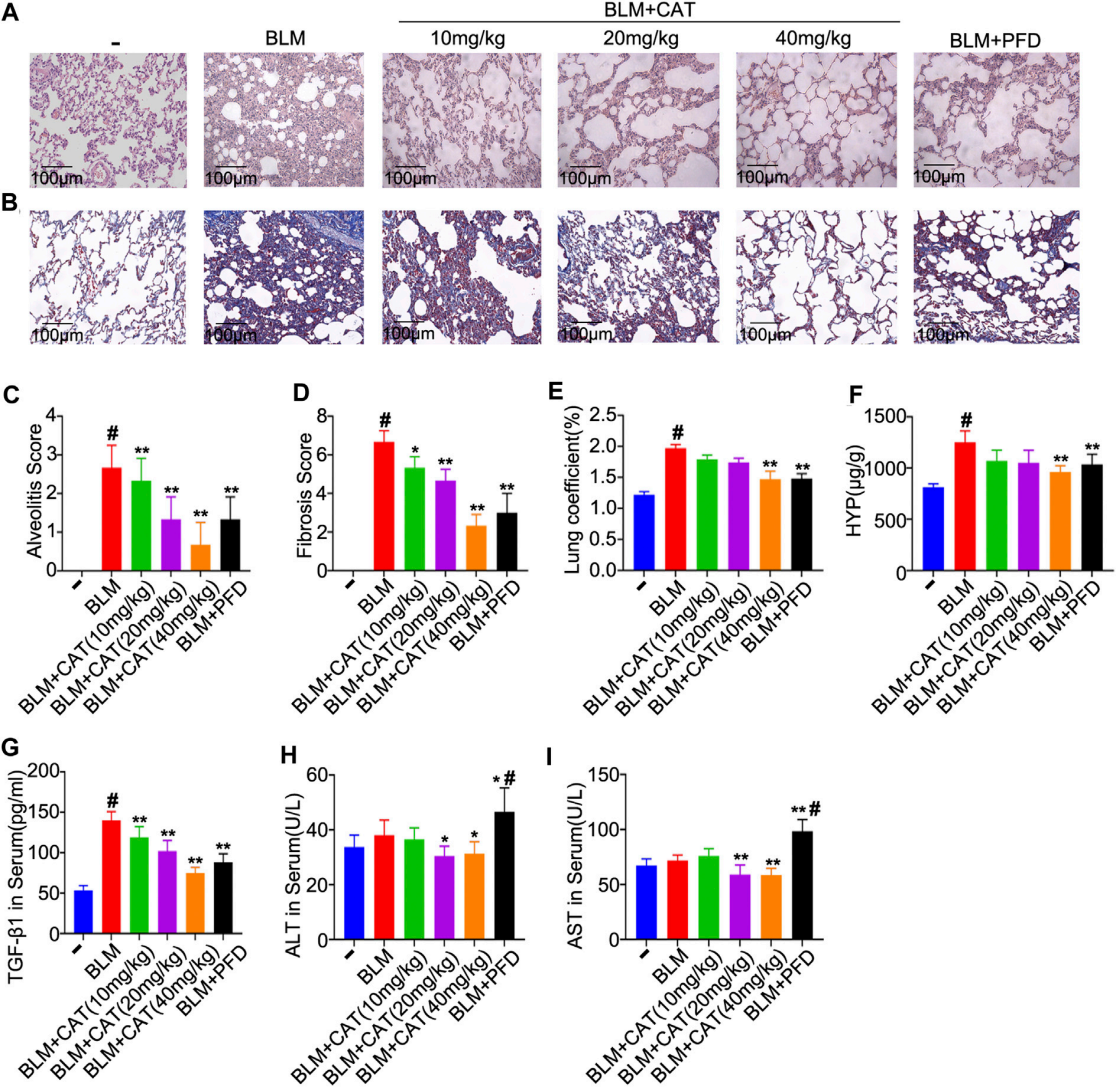
The protective effects of CAT on BLM-induced PF in rats. **(A)** Photomicrographs of HE-stained lung sections. **(B)** Photomicrographs of Masson Trichrome stained lung sections. **(C)** Alveolitis score of each group. **(D)** Statistics of pulmonary fibrosis area in each group. **(E)** Measurement of lung coefficient in each group. **(F)** Determination of HYP level in lung tissues of each group. **(G)** Determination of serum TGF-β1 levels of each group. **(H)** Determination of serum ALT levels of each group. **(I)** Determination of serum AST levels of each group. Data are presented as the means ± SD (n = 3 or 6), # represents a comparison with the control group, * represents a comparison with the BLM group. #*p* < 0.01; *p < 0.05; **p < 0.01.

**FIGURE 5:**
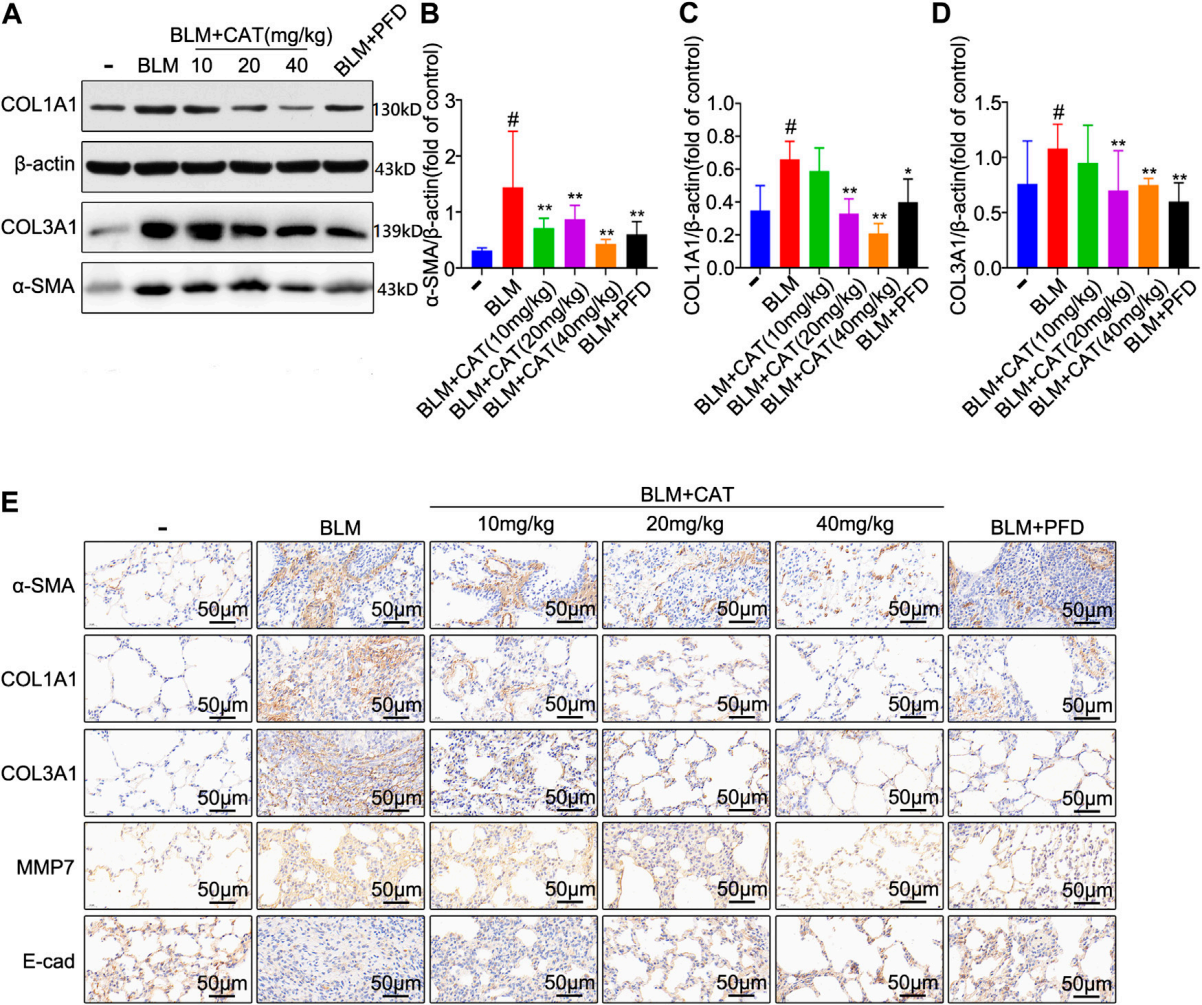
CAT attenuates BLM-induced EMT in lung tissues and ECM deposition *in vivo*. **(A)** Western blot analysis of the protein levels of α-SMA, COL1A1, and COL3A1 in lung tissues. **(B–D)** Densitometric analysis of α-SMA, COL1A1, and COL3A1 band intensities in immunoblots, using β-actin as the internal reference. **(E)** Immunohistochemical staining of α-SMA, COL1A1, COL3A1, MMP-7, and E-cad positive cells in the lungs. Data are presented as the means ± SD (n = 3), # represents a comparison with the control group, * represents a comparison with the BLM group. #*p* < 0.01; *p < 0.05; **p < 0.01.

The 2D structure of CAT (PubChem CID: 91520, CAS No. 2415-24-9) is shown in Figure 1B. PF was successfully induced by intratracheal instillation of BLM (5 mg/kg) in rats. HE staining confirmed that the structure of rat lung tissues in the BLM group was disordered, with alveolar wall thickening, infiltration of a large number of inflammatory cells in the alveolar and the interstitial cavities, and the disappearance of some alveoli. All rats survived until the samples were collected. In the three days after modeling, the weight of all rats decreased, after which it gradually increased. Compared with those in the NS group, all rats administered BLM had different degrees of wheezing, coughing, and bradykinesia, and Masson trichrome staining revealed extensive collagen deposition (Figures 4A–D). However, CAT significantly improved lung tissue structural damage caused by BLM. The protective effect of the 40 mg/kg dose was better than that of 150 mg/kg PFD, and the lung coefficient was significantly reduced in a dose-dependent manner (Figure 4E). HYP is the main component of collagen, and TGF-β1 can induce fibroblasts to synthesize a large amount of collagen. After treatment with CAT at doses of 10–40 mg/kg, HYP levels in the lung tissues of rats with lung fibrosis and TGF-β1 levels in the serum were reduced compared to those in the BLM group (Figures 4F,G). There was no significant difference between the 40 mg/kg CAT group and 150 mg/kg PFD groups, and no impairment of liver functions was observed (Figures 4H,I). E-cad and α-SMA are considered biomarkers of epithelial cells and myofibroblasts, respectively. During EMT, the expression of E-cad decreases, while the expression of α-SMA increases significantly. In addition, the EMT process in lung tissues is accompanied by collagen deposition and the activation of MMPs in the ECM. As shown in Figures 5A–D, the WB results demonstrated increased expression of α-SMA, COL1A1, and COL3A1 in the BLM group, which was also evidenced by immunohistochemistry (Figure 5E). However, CAT dramatically reversed the upregulation of these proteins and attenuated the expression of MMP-7 and the downregulation of E-cad (Supplementary Figure S7), with a better effect than that of 150 mg/kg PFD. These results suggest that CAT may be alleviating PF by inhibiting the EMT process to reduce ECM deposition.

### CAT Alleviates PF by Inhibiting the TGF-β1/Smad3 and Wnt/β-Catenin Signaling Pathways

**FIGURE 6:**
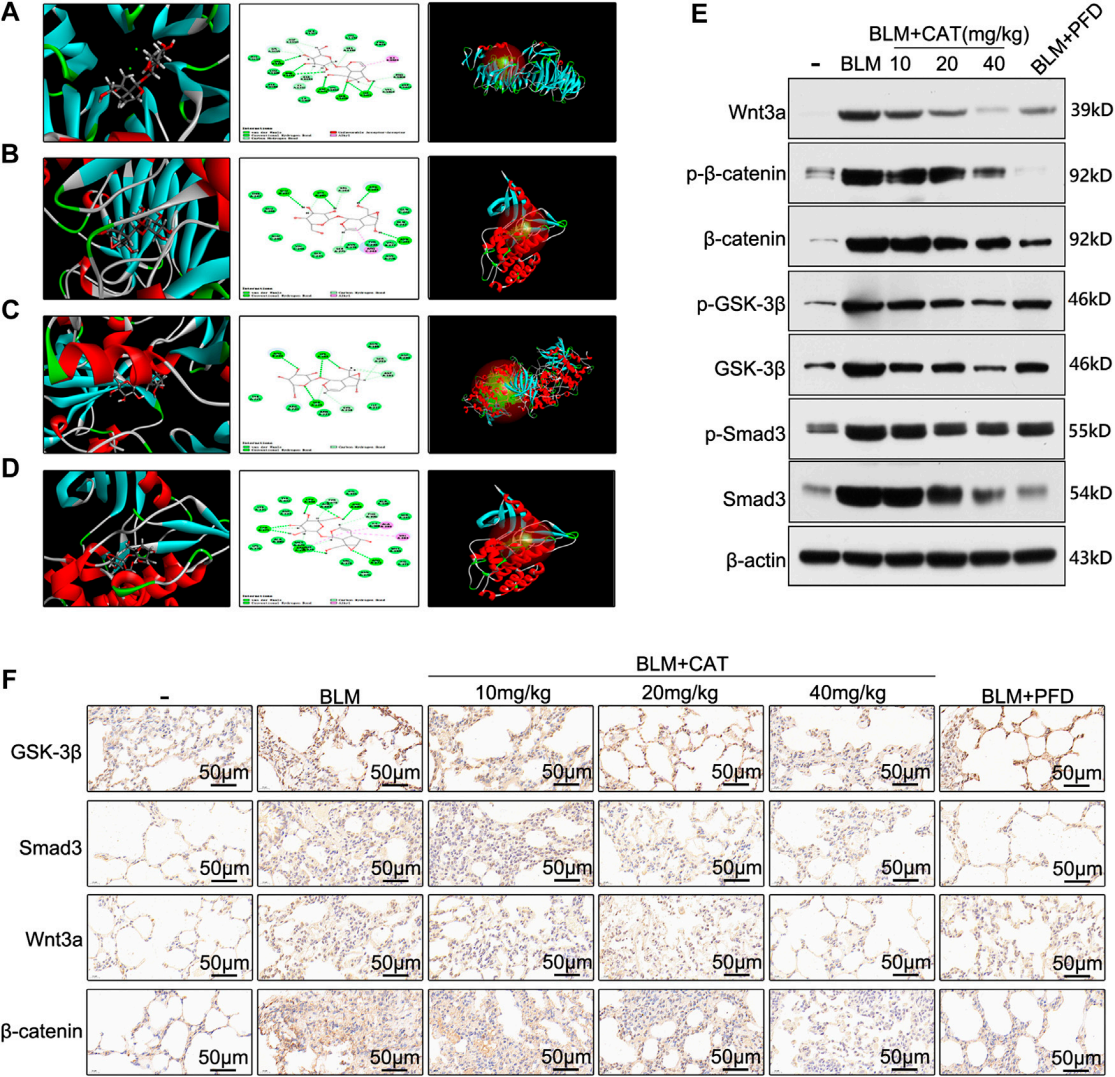
CAT alleviates PF by inhibiting the TGF-β1/Smad3 and Wnt/β-catenin signaling pathways. The docking conformation and main active site of CAT and Wnt3a **(A)**, Smad3 **(B)**, GSK-3β **(C)**, and TGF-βR1 **(D)**. **(E)** WB analysis of the protein levels of Wnt3a, p-β-catenin, β-catenin, *p*-GSK-3β, GSK-3β, *p*-Smad3, and Smad3 in lung tissues.**(F)** Immunohistochemical staining of Wnt3a, Smad3, GSK-3β, and β-catenin positive cells in the lungs. Data are presented as the means ± SD (n = 3), # represents a comparison with the control group, * represents a comparison with the BLM group. #*p* < 0.01; *p < 0.05; **p < 0.01.

In order to determine the mechanism of action of CAT in IPF, we used molecular docking technology to dock key proteins from the PPI network analysis and KEGG pathway analysis with CAT. The CDOCKER results revealed that CAT can be docked effectively with Wnt3a (PDB code: 4A0P), Smad3 (PDB code: 1U7F), GSK-3β (PDB code: 4J1R), and TGF-βR1 (PDB code: 6B8Y) (binding energies: 57.42 kcal/mol, 48.22 kcal/mol, 36.10 kcal/mol, and 23.75 kcal/mol, respectively). CAT forms an alkyl interaction with residue ILE1023 of Wnt3a; Van der Waals bonds with residues VAL1194, PRO974, ASP1022, VAL1063, CA1360, ALA1108, LEU1109, PHE1153, and SER1111; hydrogen bonds with residues VAL1152, THR1151, ASP971, SER1020, and ILE1021; and carbon-hydrogen bonds with residues HIS1192, PRO1066, VAL1064, ALA1193, CL1355, LYS1195, ASP1110. It forms alkyl interactions with residue ARG243 of Smad3; van der Waals bonds with residues GLN242, VAL277, ASN278, TYR238, ASN276, GLY245, VAL244, GLU246, and THR247; hydrogen bonds with residues ARG243, ASN241, GLU337, and GLY245; and carbon-hydrogen bonds with residues VAL244, and SER275. It forms van der Waals bonds with residues ASN186, ASP200, ILE217, ARG223, ARG220, and TYR221 of GSK-3β; hydrogen bonds with residues ARG220, LYS183, and SER219; and carbon-hydrogen bonds with residues SER203, ASP181, and CYS218. It forms alkyl interactions with residues ALA399 and VAL383 of TGF-βR1; van der Waals bonds with residues HIS331, ALA330, SER395, ILE388, MET390, HIS371, ASN370, VAL373, GLY374, MET379, ALA380, LYS376, LYS335, ASP333, TYR402, and ALA403; hydrogen bonds with residues LEU334, ASP400, ARG372, ARG332, and THR375; and carbon-hydrogen bonds with residues TYR378 and PHE396 (Figures 6A–D). After BLM (5 mg/kg) successfully established the PF rat model, the expression of Smad3, Wnt3a, β-catenin, and GSK-3β were all upregulated in the BLM group (*p* < 0.01). After PFD and different doses of CAT were administered, the expression of Smad3, Wnt3a, β-catenin, and GSK-3β decreased in a dose-dependent manner (*p* < 0.05) (Figure 6E). The immunohistochemistry results also reflected this (Figure 6F). It was found that BLM induced the phosphorylation of β-catenin, GSK-3β, and Smad3 in the rat model, while p-β-catenin, *p*-GSK-3β, and *p*-Smad3 levels were significantly downregulated by CAT at doses of 20 mg/kg and 40 mg/kg (Supplementary Figure S8). PFD downregulated p-β-catenin and *p*-Smad3 levels, but had no significant effect on *p*-GSK-3β level. In conclusion, the TGF-β1/Smad3 and Wnt/β-catenin signaling pathways were activated in the lung tissue of rats with PF. CAT downregulated the expression of Wnt3a, β-catenin, GSK-3β, and Smad3 in a dose-dependent manner, and inhibited the phosphorylation of β-catenin, GSK-3β, and Smad3.

### CAT Reduces Oxidative Stress and Inflammation in Lung Tissue of Rats With PF

**FIGURE 7:**
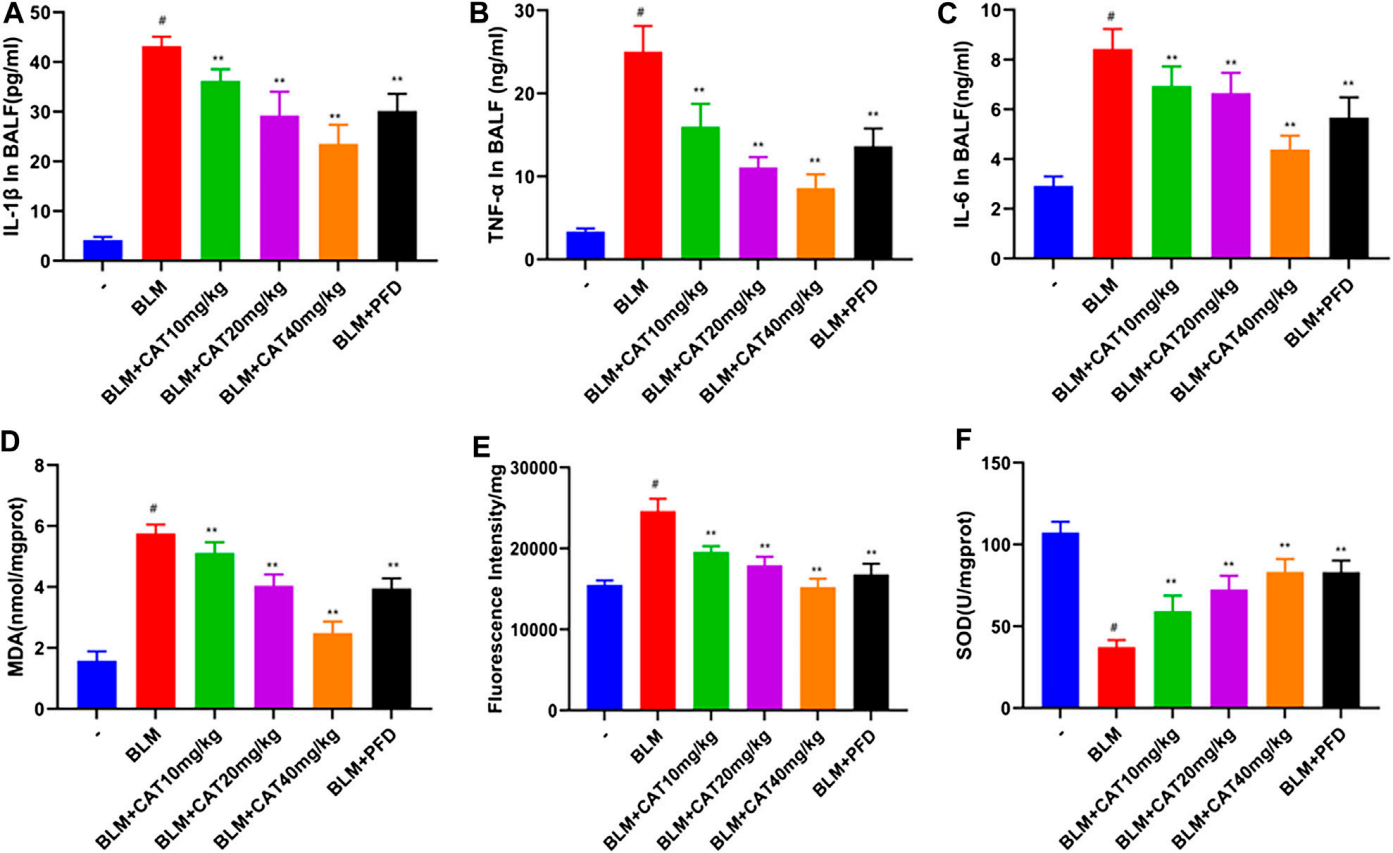
CAT reduces oxidative stress and inflammation in lung tissue of rats with PF. **(A)** Determination of IL-1β levels in BALF of each group. **(B)** Determination of TNF-α levels in BALF of each group. **(C)** Determination of IL-6 levels in BALF of each group. **(D)** Determination of MDA levels in lung tissue of each group. **(E)** Determination of ROS levels in lung tissue of each group. **(F)** Determination of SOD levels in lung tissue of each group. Data are presented as the means ± SD (n = 6); # represents a comparison with the control group, * represents a comparison with the BLM group. #*p* < 0.01; *p < 0.05; **p < 0.01.

TNF-α induces activation of the NF-κB signaling pathway and exacerbates PF caused by BLM(Hou et al., 2018). To verify the anti-inflammatory and anti-oxidant effects of CAT, we used ELISA to detect the relevant biomarkers. As expected, 28 days after successful modeling, the levels of inflammatory mediators and oxidative stress markers in the lung tissue of rats in the BLM group were increased, while CAT reduced the levels of IL-1β (Figure 7A), TNF-α (Figure 7B), IL-6 (Figure 7C), MDA (Figure 7D), and ROS (Figure 7E) and increased the activity of SOD (Figure 7F) in the lung tissues of IPF rats. The CAT and PFD at 40 mg/kg were significantly different from BLM groups. In summary, CAT can downregulate the expression of cytokines in lung tissues and reduce the level of oxidative stress, thus alleviating BLM-induced PF in rats.

## Discussion

IPF is an irreversible, progressive, and fatal lung disease with a poor prognosis that often occurs during acute exacerbation (Border and Noble, 1994; Lopez et al., 2009). Since the molecular mechanisms underlying the occurrence and development of PF are not fully understood, the treatments approved so far are limited to those effective against mild-to-moderate IPF(Mora et al., 2017). The pathogenesis of IPF involves a variety of processes, such as inflammation, oxidative stress, and fibrosis. In this study, we discovered that 141 upregulated genes and 25 downregulated genes were common between the three IPF data sets analyzed. GO analysis of the DEGs revealed that they were mainly involved in humoral immunity, collagen metabolism, epithelial cell proliferation, mesenchymal development, and cell matrix adhesion, which is consistent with the strong inflammatory response and the imbalance of collagen metabolism observed in IPF (Xin et al., 2019). KEGG pathway enrichment analysis showed significant enrichment in protein degradation and absorption, cytokine-cytokine receptor interaction, and ECM-receptor interaction, all of which are areas most current studies on IPF are focused on (Du et al., 2019). Although hypoxia is a significant pathological feature of PF (Senavirathna et al., 2018)—for example, the nuclear HIF-1α protein is involved in hypoxia-induced EMT (Senavirathna et al., 2018)—several key protein targets for cellular hypoxia as well as oxidative stress did not appear in the PPI results. Correspondingly, the support for the GO analysis and KEGG pathway enrichment analysis is not high. Although the reasons for these differences are not clear, we suggested that researchers should re-examine the importance of the known key signaling pathways and biological processes in the development of IPF.

Traditional Chinese medicine is a potential source of treatments for PF (Li and Kan, 2017). We performed molecular docking of CAT, the active constituent of *Rehmannia glutinosa*, with several key protein targets suggested by the PPI and KEGG enrichment analyses and found that CAT has high binding to several components of the TGF-β1/Smad3 and Wnt/β-catenin pathways. TGF-β1 is a key protein among the many factors and cytokines that regulate PF (Border and Noble, 1994). It can induce alveolar epithelial cells to acquire the phenotype of mesenchymal cells, which become the main source of fibroblasts and myofibroblasts and lead to ECM deposition (Song et al., 2013). This process, as a typical EMT, is a very important mechanism of PF (Piera-Velazquez et al., 2011), and had a high score in GO analysis and KEGG pathway enrichment analysis. Specifically, TGF-β1 interacts with fibroblast surface receptors and phosphorylates Smad proteins, which form Smad3/4 complexes. These enter the nucleus and bind to the promoter regions of fibrogenic genes, such as those for type I collagen, fibronectin, and α-SMA, to activate downstream target gene transcription (Santibanez et al., 2011). However, although TGF-βR1-mediated receptor-activated Smad proteins are most closely related with the occurrence of PF(Ask et al., 2008) and can participate in the regulation of EMT through multiple pathways, the Wnt/β-catenin signaling pathway is even more critical for mediating EMT. It is a key pathway regulating cell proliferation and differentiation, with β-catenin as its major signal transduction molecule. Trauma and other stimuli activate the expression and secretion of the Wnt protein, which leads to the inhibition of β-catenin phosphorylation (Li et al., 1999). Once a certain amount of free β-catenin accumulates, it enters the nucleus and activates target gene transcription (Nusse and Clevers, 2017). The epithelial phenotypic marker E-cad is downregulated and the myofibroblast markers α-SMA and type I collagen are upregulated in PF (Qu et al., 2015; Lacy et al., 2018), and the high expression of α-SMA is associated with a lower survival rate in patients with PF. As targets gene for β-catenin activation, the expression of MMPs accelerates the degradation of ECM. MMPs are the main rate-limiting enzymes that regulate ECM metabolism. MMP-7, which is able to activate other proteases while degrading ECM components such as cell-associated Fas ligands and E-cad, plays a key role in regulating various cell processes such as matrix remodeling, apoptosis, and EMT (Zhou et al., 2017). Wnt/β-catenin signaling pathway is a key factor in the regulation of MMP-7 *in vivo*. The activation of β-catenin promotes the expression of MMP-7 (He et al., 2012; Zuo and Liu, 2018). Thus, it can be seen that the Wnt/β-catenin pathway plays a significant role in the process of PF.

In recent years, several studies have shown that there is crosstalk between the TGF-β/Smad3 and Wnt/β-catenin signaling pathways. Axin and GSK-3β in the Wnt/β-catenin pathway can affect TGF-β signaling by controlling the stability of Smad3 (Guo et al.,2008), while Smad3-mediated regulation enhances the stability of β-catenin and promotes the activation of downstream target genes (Zhang et al., 2010). In addition, p-β-catenin/p-Smad2 complexes were also found in the lung tissues of patients with PF (Kim et al., 2009), while Wnt3a/β-catenin/GSK-3β were mainly localized in alveolar and bronchial epithelial cells (Konigshoff et al., 2008). Therefore, targeting the TGF-β1/Smad3 and Wnt/β-catenin signaling pathways is an effective strategy for regulating EMT and inhibiting the progression of PF. *In vivo* experiments showed that the TGF-β/Smad3 and Wnt/β-catenin pathways were activated in a rat model of PF (Henderson et al., 2010; Kim et al., 2011), which was consistent with previous studies. Molecular docking, WB, and immunohistochemistry experiments confirmed that CAT can bind to Wnt3a, Smad3, GSK-3β, and TGF-βR1. Furthermore, by inhibiting the activation of the TGF-β/Smad3 and Wnt/β-catenin pathways, CAT reduced the secretion of key signal transduction molecules such as Smad3, Wnt3a, GSK3β, and β-catenin, and also reduced the expression of α-SMA, COL1A1, COL3A1, and MMP7, effectively inhibited EMT, ECM deposition, and lung structural remodeling and reduced the downregulation of the epithelial phenotype marker E-cad. This demonstrates that CAT can maintain the balance of ECM degradation in the local lung microenvironment, which is of great significance for improving outcomes in PF.

As IPF occurs, fibroblasts and myofibroblasts proliferate extensively in fibroblast foci. Factors such as CXCL12 are released by damaged alveolar epithelial cells and cause CXC chemokine type 4 receptor-positive circulating fibroblasts to enter the lungs, expanding the fibroblast pool (Andersson-Sjoland et al., 2008; Mehrad et al., 2009). At the same time, alveolar epithelial cells entering the phenotypic state associated with aging produce and release a large number of cytokines such as TNF-α. Moreover, high inflammatory levels also cause local fibroblasts to migrate and hyperproliferate, and oxidative stress can also lead to the release of pro-inflammatory factors such as IL-1β, which plays an important role in the pathogenesis of IPF (Kliment et al., 2009). Oxidative stress is essentially the imbalance between oxidation and reduction caused by excessive generation of ROS in the body (Kala et al., 2017). SOD is an important antioxidant enzyme that maintains the dynamic balance between free radical generation and removal (He et al., 2016). BLM can cause alveolar epithelial cells and macrophages to produce a large amount of ROS, resulting in lipid peroxidation in biofilms, while MDA can reflect the degree of cell damage by reflecting lipid peroxidation (Teixeira et al., 2008; Yu et al., 2016). This study revealed that oxidative stress and inflammation levels were elevated in rats with PF, which is consistent with previous findings (Xin et al., 2019). With reference to the results of the bioinformatics analysis, *in vivo* experiments confirmed that CAT could increase the activity of SOD in rat lung tissues and decrease the levels of MDA, IL-6, IL-1β, TNF-α, and ROS, suggesting that CAT may inhibit the development of PF by reducing the levels of inflammation and oxidative stress. Although our results prove that CAT has a potential therapeutic effect on BLM-induced pulmonary fibrosis in rats, the more detailed mechanism of action is still unclear. For example, how does CAT inhibit β-catenin activation/phosphorylation in the Wnt signaling pathway? One possible explanation is that CAT blocks the interaction between Wnt3a and β-catenin. In addition, if we use primary human embryonic lung fibroblasts for *in vitro* experiments, our findings will be more meaningful.

## Conclusion

In summary, the results of this study suggest that the collagen metabolism imbalance, inflammatory responses, and EMT activation are the core processes of IPF, and that the TGF-β1/Smad3 and Wnt/β-catenin signaling pathways and related signal transduction molecules are key targets for the treatment of IPF. The ability of CAT to protect against lung fibrosis induced by BLM in rats was reported for the first time. This mechanism is related to the downregulation of Smad3, Wnt3a, GSK-3β, and β-catenin as well as the phosphorylation of Smad3, GSK-3β, and β-catenin. This study also provided new insights into the potential value of CAT for the treatment of IPF. Considering that CAT did not harm the liver during this study and that there have been previous studies on CAT in combination with other compounds to reduce drug-induced hepatitis, further research should focus on clinically evaluating the effectiveness and side-effects of CAT in patients with PF.

## Data Availability Statement

The datasets presented in this study can be found in online repositories. The names of the repository/repositories and accession number(s) can be found in the article/Supplementary Material.

## Ethics Statement

The animal study was reviewed and approved by Research Ethics Committee of the Affiliated Hospital of Shandong University of Traditional Chinese Medicine.

## Author Contributions

FY, WZ, and WL conceived and designed the experiments; ZH, HZ, and XC performed the experiments; RC, YL contributed reagents/materials/analysis tools; FY and RC analyzed the data and wrote the paper. All authors have read and agreed to the published version of the manuscript.

## Funding

This work was supported by the National Natural Science Foundation of China (Grant No. 81874442), the Taishan Scholars Program of Shandong Province in China of Pulmonary disease of traditional Chinese Medicine (Grant No. ts201712096) and the innovative research program for graduates of Central South University (No. 2020zzts807).

## Conflict of Interest

The authors declare that the research was conducted in the absence of any commercial or financial relationships that could be construed as a potential conflict of interest.

## Abbreviations

BALF, bronchoalveolar lavage fluid; BLM, Bleomycin; CAT, Catalpol; DAVID, The database for Annotation, Visualization and Integrated Discovery; DEGs, differentially expressed genes; ECM, extracellular matrix; EMT: epithelial-mesenchymal transition; GEO, Gene Expression Omnibus database; ILDs, interstitial lung diseases; IPF, idiopathic pulmonary fibrosis; KEGG, Kyoto Encyclopedia of Genes and Genomes; PF, pulmonary fibrosis; PFD, Pirfenidone; PPI, protein-protein interaction.
