# Supplementary figures and images for: Catalpol Protects Against Pulmonary Fibrosis Through Inhibiting TGF-β1/Smad3 and Wnt/β-Catenin Signaling Pathways
Source: Front Pharmacol. 2021 Jan 29;11:594139. doi: 10.3389/fphar.2020.594139 (PMC7878558; doi:10.3389/fphar.2020.594139)

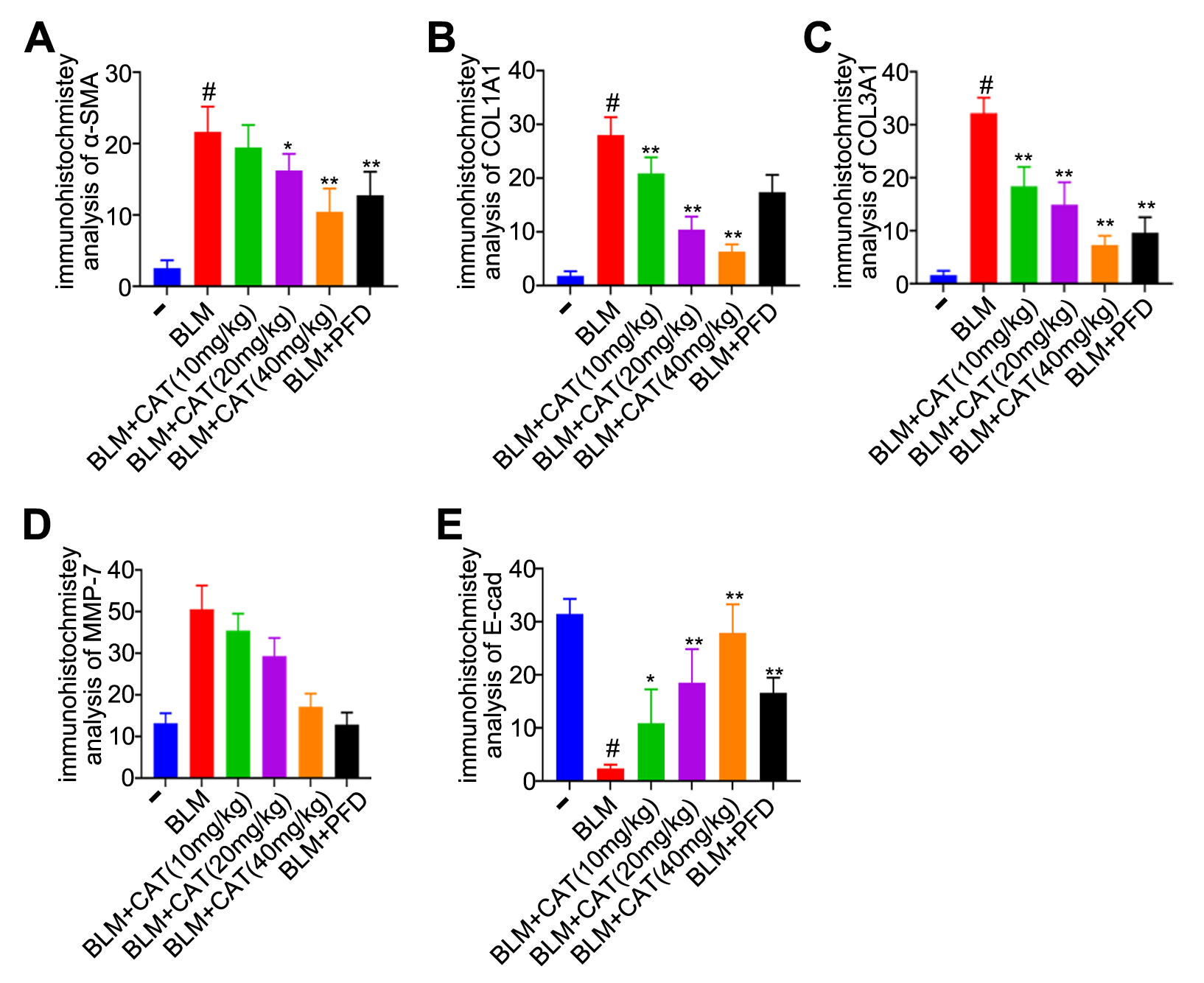

Supplement: Supplementary file 1 [file image1.tif]

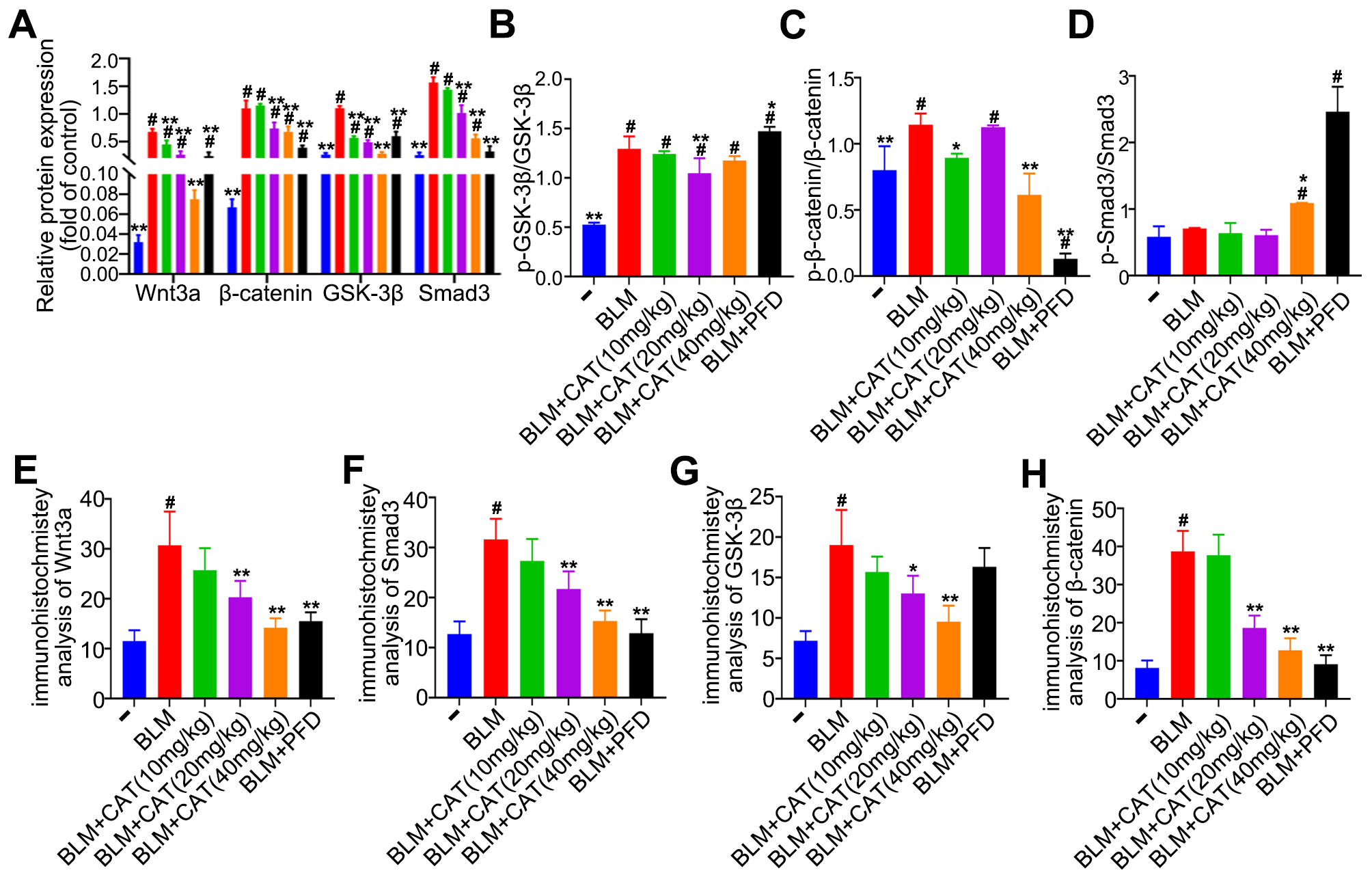

Supplement: Supplementary file 2 [file image2.tif]

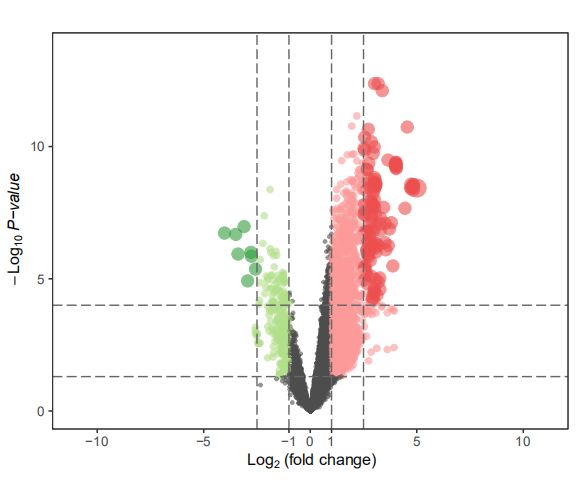

Supplement: Supplementary file 3 [file image3.png]

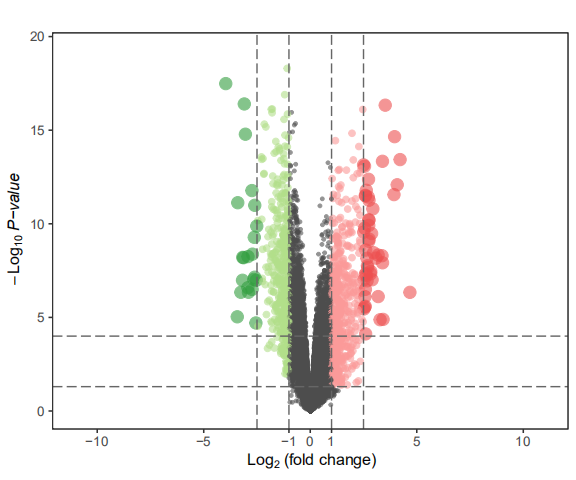

Supplement: Supplementary file 4 [file image4.png]

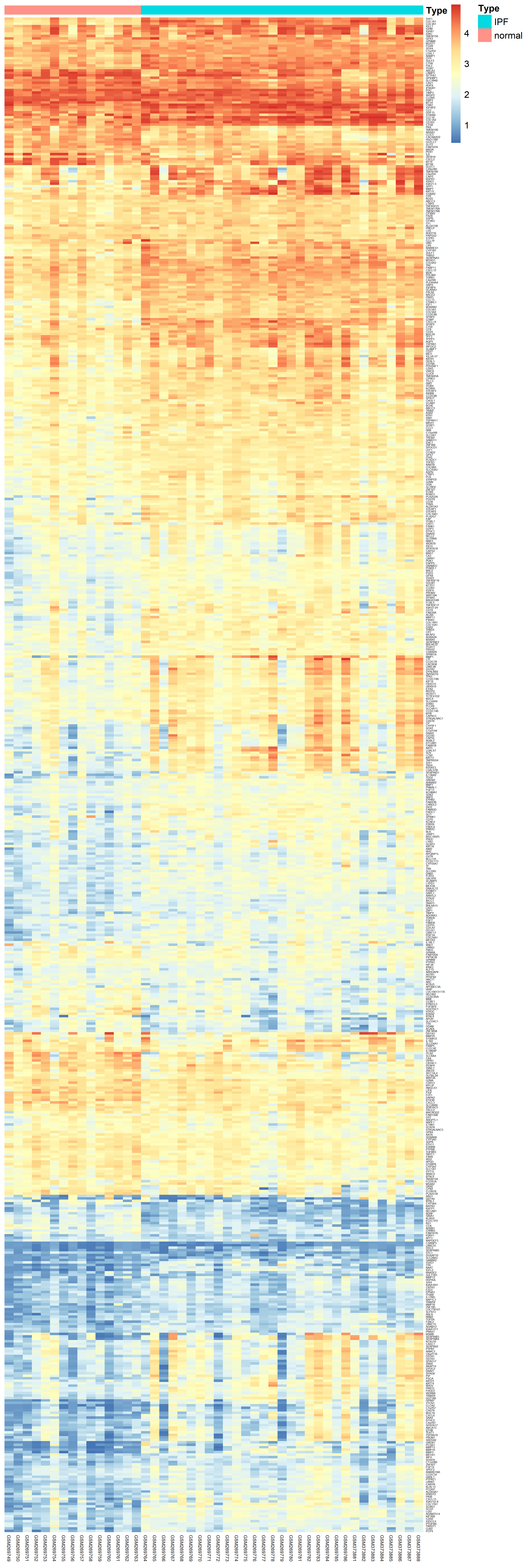

Supplement: Supplementary file 5 [file image5.tiff]

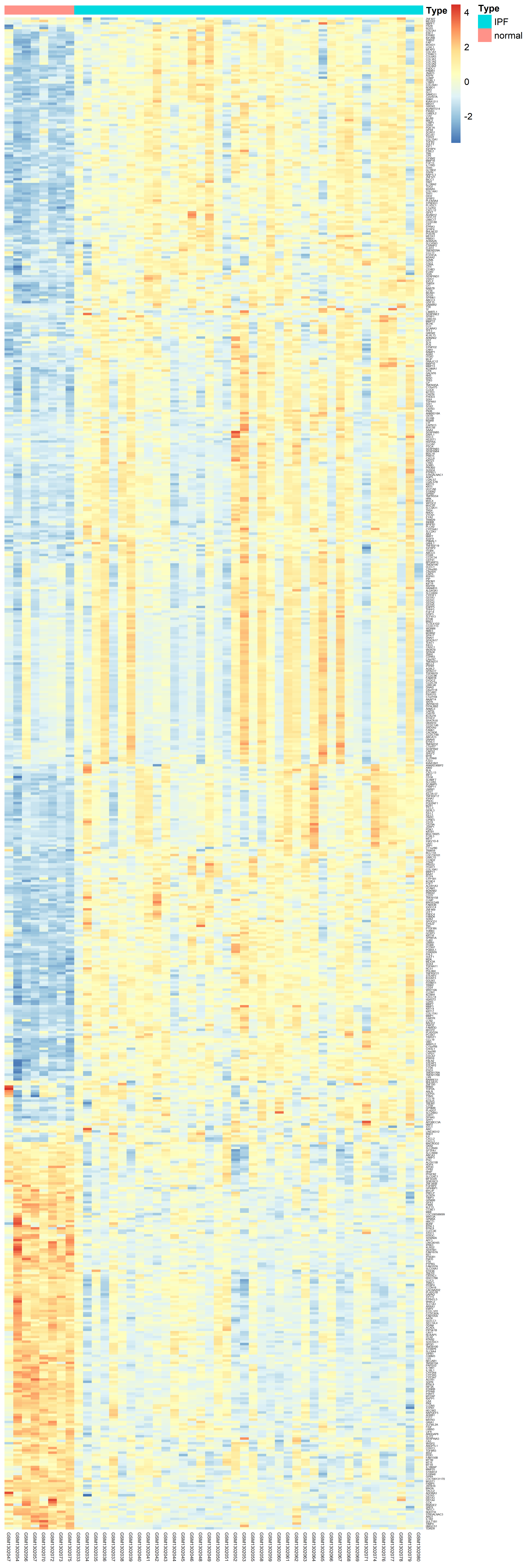

Supplement: Supplementary file 6 [file image6.tiff]

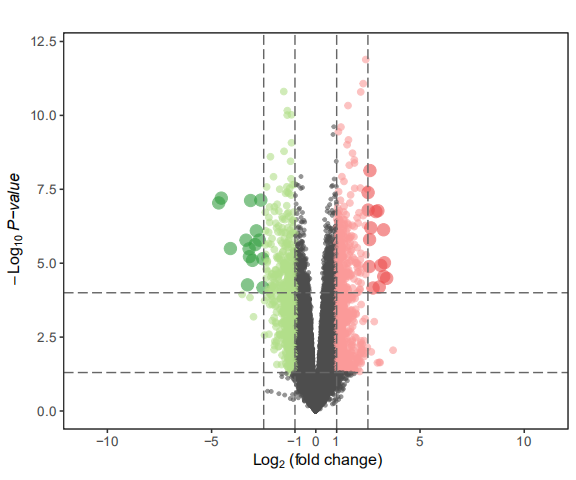

Supplement: Supplementary file 7 [file image7.png]

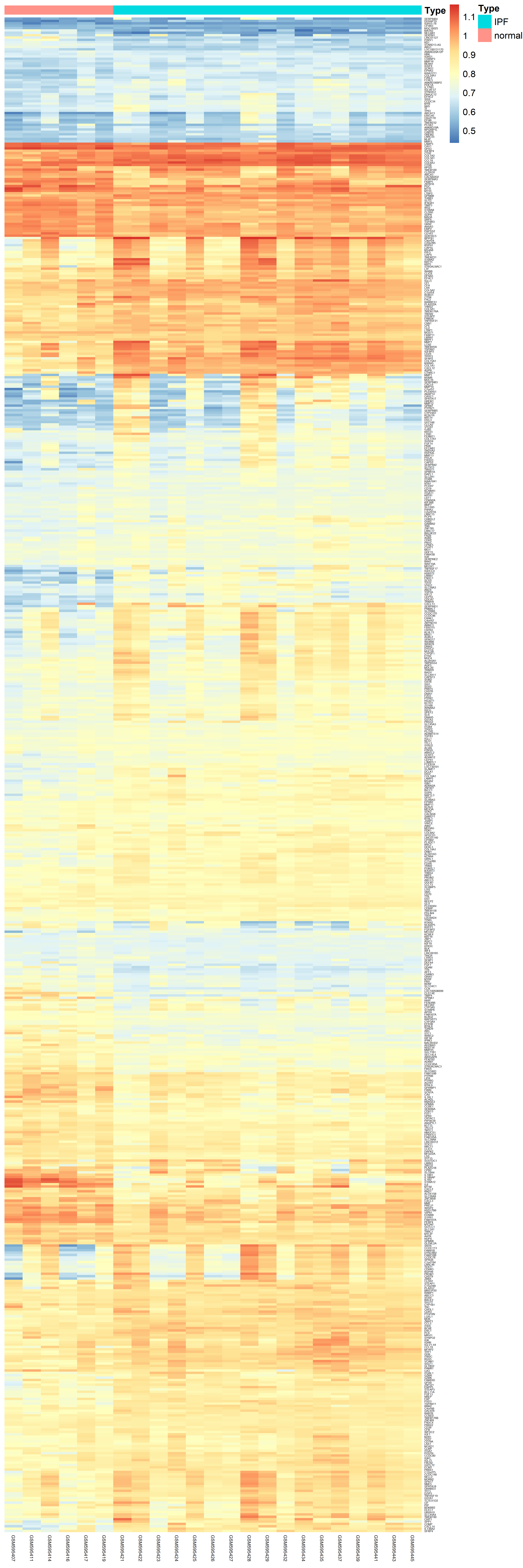

Supplement: Supplementary file 8 [file image8.tiff]
